# Oncology nurse navigator core competencies and patient satisfaction: A cross-sectional correlational study at an Egyptian cancer center

**DOI:** 10.1097/MD.0000000000050867

**Published:** 2026-09-25

**Authors:** Hasan Ahmed Awad Bassyouny, Hossam Ali Ismail, Aliaa Ezz Eldin Abd Elmoaty, Mohamed Ahmed Aly, Rasha Kadri Ibrahim, Abdelaziz Hendy, Wafa Almegewly, Rasha Mohammed Hussien, Samar Mahmoud Mohamed Elhadary, Mohamed Hashem Kotp

**Affiliations:** a Nursing Administration, Faculty of Nursing, Helwan University, Cairo, Egypt; b Department of Nursing, College of Applied Medical Sciences, King Faisal University, Al Ahsa, Saudi Arabia; c Prince Sattam Bin Abdulaziz University, Wadi Addawasir, Saudi Arabia; d Nursing Department, Fatima College of Health Sciences, Madinat Zayed, UAE; e Faculty of Nursing, Ain Shams University, Cairo, Egypt; f Department of Maternal and Child Health Nursing, College of Nursing, Qassim University, Buraydah, Saudi Arabia; g Department of Community and Psychiatric Mental Health Nursing, College of Nursing, Princess Nourah bint Abdulrahman University, Riyadh, Saudi Arabia; h Department of Community, Psychiatric, and Mental Health Nursing, College of Nursing, Qassim University, Buraydah, Qassim, Saudi Arabia; i Pediatric Nursing, Faculty of Nursing, Cairo University, Cairo, Egypt; j College of Nursing at Gulf Medical University in Ajman, UAE; k Nursing Department, Fakeeh College for Medical Sciences, Fakeeh Care Group, Jeddah, Saudi Arabia.

**Keywords:** cancer, clinical competence, oncology nursing, patient navigation, patient satisfaction, patient-centered care

## Abstract

Oncology nurse navigators (ONNs) are increasingly recognized for their vital role in improving the quality of cancer care by supporting patients throughout their treatment journey. In Egypt, nurse navigation implementation remains limited, and empirical evidence examining the relationship between ONN competencies and patient satisfaction is scarce. This study aimed to assess the core competencies of ONNs and their relationship to patient satisfaction at a leading cancer center in Egypt. A descriptive correlational study was conducted among 65 ONNs and 404 oncology patients at a major oncology hospital in Cairo. Data were collected using 2 previously developed instruments: the ONN Core Competencies Questionnaire and the Patient Satisfaction with the Navigator Scale. The mean total ONN competency score was 79.28 ± 8.24, and the mean patient satisfaction score was 93.18 ± 5.68. At the nurse-navigator level, overall ONN competency was strongly and positively associated with navigator-level mean patient satisfaction (*r* = .70, 95% CI [.55–.81], *P* < .001). All competency domains were positively associated with mean patient satisfaction, with the strongest domain-level associations observed for Expert ONN competency (*r* = .65, 95% CI [.50–.78]) and coordination of care (*r* = .56, 95% CI [.37–.71]). Competency scores differed significantly according to nurse age, education, experience, and training frequency, whereas patient satisfaction differed significantly according to patient age and gender. Higher ONN competency scores were associated with higher navigator-level mean patient satisfaction, particularly for expert navigation and care coordination competencies. These findings support continued professional development and strengthening of oncology nurse navigation services; however, the observed associations are nurse-level and cross-sectional and should not be interpreted as individual-patient-level or causal effects.

## 1. Introduction

Oncology nursing has evolved in response to the growing cancer burden and the increasing complexity of cancer care. Oncology nurses contribute to clinical management, psychosocial support, patient education, care coordination, and continuity of care across the cancer trajectory.^[1]^ In low- and middle-income countries, including Egypt, the development of specialized oncology nursing roles remains constrained by limited training opportunities, scarce resources, and workforce shortages.^[2,3]^ Nevertheless, rising demand for high-quality cancer care has spurred efforts to strengthen oncology nursing education, infrastructure, and policy support.^[4]^

The specialization of oncology nursing has led to roles such as oncology nurse practitioners, palliative care nurses, and oncology nurse navigators (ONNs). ONNs help patients navigate complex healthcare systems, access timely care, and overcome barriers throughout diagnosis, treatment, and survivorship.^[5–10]^ ONN core competencies include care coordination, communication, education, professional practice, and expert navigation.^[11–13]^ These competencies require knowledge of cancer care and healthcare systems, effective communication and advocacy skills, and patient-centered attributes such as empathy and cultural sensitivity.^[14–16]^

ONNs support transitions across phases of cancer care, facilitate access to services, provide information on disease and treatment, and coordinate communication between patients and multidisciplinary teams.^[17–21]^ They also help identify barriers related to transportation, finances, language, health literacy, employment, insurance, and referrals.^[14,22–24]^ Through collaboration with cancer committees and healthcare managers, ONNs may contribute to service planning, program evaluation, community needs assessment, and quality improvement.^[25,26]^ Their advocacy and coordination roles are particularly important for ensuring that patients’ needs and preferences are communicated to the healthcare team.^[27–29]^

Patient satisfaction is a key indicator of healthcare quality, particularly in oncology, where patients often face complex treatment pathways and substantial informational and psychosocial needs.^[30]^ Nurse navigation services provide continuous guidance and support that may help patients feel informed, empowered, and less overwhelmed throughout their care journey.^[31,32]^ Evidence links nurse navigation to shorter intervals between diagnosis and treatment, improved communication, greater treatment adherence, and higher patient satisfaction.^[33–35]^ Patients receiving navigation support have reported more positive care experiences, greater satisfaction with cancer care, improved knowledge and self-efficacy, and fewer scheduling difficulties.^[36–38]^

However, evidence on the broader effects of nurse navigation is inconsistent. Although some studies have reported higher satisfaction and self-efficacy among patients receiving navigation services, improvements in health-related quality of life and other clinical outcomes have been less consistent.^[39,40]^ These findings suggest that patient satisfaction may be particularly relevant when evaluating nurse navigation services. Nevertheless, limited evidence has examined whether ONN professional competencies are associated with patient satisfaction, particularly in healthcare settings where nurse navigation remains underdeveloped.

Despite growing recognition of the ONN role, a substantial evidence gap remains in Egypt, where few studies have examined the link between ONN competencies and patient satisfaction. Understanding this link may inform the development, evaluation, and professional preparation of oncology navigation services. Therefore, this study aimed to assess ONNs’ core competencies and examine their association with patient satisfaction at an Egyptian cancer center.

### 1.1. Research questions

1. What are the current competency levels of nurse navigators?
2. How satisfied are patients with the role of ONNs?
3. What is the correlation between ONN competencies and patient satisfaction?

## 2. Methods

### 2.1. Research design and setting

This study employed a descriptive-correlational design to examine the relationship between ONN competencies and patient satisfaction. This design was chosen to explore existing patterns and associations in this under-researched area and provide a comprehensive understanding of the dynamics among these variables. The study was conducted at a prominent oncology hospital in Cairo, Egypt, selected for its established patient navigation program, which provided an appropriate setting for examining this relationship.

### 2.2. Subjects

A census sampling approach was used to recruit ONNs. All 65 registered nurses working in the oncology navigation program at the selected hospital in Cairo, Egypt, who met the eligibility criteria, were invited to participate in the study. Nurses were eligible if they had at least 2 years of professional experience in oncology nursing and at least 6 months of experience as ONNs. Their responsibilities included coordinating patient care, addressing treatment barriers, and providing emotional and psychological support.

The patient sample consisted of 404 individuals receiving care through the hospital’s oncology navigation program. The sample size was estimated a priori using Cochran formula^[41]^ for large-population samples:


$${\text{n}}_{\text{0}}=(Z^{\text{2}}\times P\times (1-P))/{\text{e}}^{\text{2}}$$

Where: n_0_ = required sample size. *Z* = 1.96 (*Z*-score for 95% confidence level). *P* = .5 (assumed proportion for maximum variability). e = 0.05 (margin of error).


$$\begin{array}{c} {\text{n}}_{\text{0}}=(1.96{)}^{\text{2}}\times 0.5\times (1-0.5)/(0.05{)}^{\text{2}}=384.16 \end{array}$$

This calculation yielded a minimum required sample size of 385. To account for potential nonresponses, data loss, and incomplete questionnaires, an additional 19 participants were added, resulting in a final sample size of 404 patients. This oversampling strategy aligns with standard survey methodology to ensure representativeness and statistical precision.^[42]^

Furthermore, a post hoc power analysis was conducted using G*Power version 3.1.9.7 to confirm the adequacy of the correlation analysis. Based on the observed correlation coefficient (*r* = 0.70), alpha level of 0.05 (2-tailed), and the effective sample size of 65 paired observations, the calculated statistical power exceeded 0.99, indicating adequate power to detect the observed association between ONN competency scores and corresponding mean patient satisfaction scores.

Eligible patients were adults (18 years or older) who were actively involved in their treatment plans, capable of providing informed consent, and had had at least one interaction with an ONN. Simple random sampling ensured unbiased participant selection. The final response rate was 96.6%, indicating high engagement and data completeness.

The patient sample was selected via simple random sampling from the list of patients enrolled in the oncology navigation program. Eligible patients were randomly selected to participate until the target sample size was reached. This method ensured an unbiased, representative sample while remaining practical within the hospital context.

### 2.3. Tools for data collection

#### 2.3.1. Tool I: ONN Core Competencies Questionnaire

This tool was designed to evaluate ONNs’ professional competencies. It consisted of 2 parts:

*Part I: Personal data*: This section collected demographic and professional information, including age, educational level, sufficiency of monthly income, nursing experience in the oncology department, and participation in navigation-related training programs, as described by Freeman and Rodriguez^[7]^ and Bailey et al.^[43]^

*Part II: ONN Core Competencies*: This section is organized into 5 key categories: care coordination, communication, education, professional role, and advanced skills unique to expert ONNs. These competencies were previously published in earlier studies by Bailey et al^[43]^ and Jean-Pierre et al.^44]^ Each competency item was rated on a 3-point scale: “Completely Done” (2 points), “Partially Done” (1 point), or “Not Done” (0 points). The total score for each category was calculated by summing the item ratings within that category, providing a detailed assessment of navigators’ abilities to address patient needs, provide education and emotional support, and collaborate effectively within multidisciplinary care teams. Higher scores indicate higher competency.^[43,44]^ The scale comprises 52 items in total, with score ranges as follows: Total Core Competencies: 0–104 (52 items), Coordination of Care: 0–26 (13 items), Communication: 0–18 (9 items), Education: 0–22 (11 items), Professional Role: 0–14 (7 items), Expert ONN: 0–24 (12 items). The reliability of the ONN core competency scale was reported as acceptable, with a Cronbach alpha of 0.76.^[45]^

#### 2.3.2. Tool II: patient satisfaction with the Navigator Scale (PSN)

This tool was designed to measure patients’ satisfaction with the support provided by nurse navigators and consists of 2 sections:

*Part I: Personal data*: This section collected patient demographic information, including age, educational level, employment status, place of residence (rural or urban), type of cancer diagnosis, and diagnosis status (e.g., confirmed cancer, non-cancer diagnosis, or no definitive diagnosis). These details helped contextualize patients’ experiences and specific healthcare needs and were informed by foundational studies, including Wells et al^[46]^ and Esser et al.^[47]^

*Part II: PSN*: The PSN, previously published in Jean-Pierre et al,^[44]^ measures patients’ satisfaction with navigation services. The scale comprises 2 subscales:

1. *Logistical Aspects of Navigation (PSN-L*): Assesses patient satisfaction with logistical support, including appointment scheduling, understanding medical instructions, managing financial concerns, and transportation access. Responses are scored as “Very Satisfied” (3 points), “Little Satisfied” (2 points), “Not Satisfied” (1 point), and “Not a Problem” (0 points).^[44]^
2. *Interpersonal Relationship with the Navigator (PSN-I*): Assesses the quality of the patient-navigator relationship, emphasizing ease of communication, dependability, respectfulness, and empathy. Items are rated on a 5-point Likert scale, ranging from “Strongly Agree” (5 points) to “Strongly Disagree” (1 point).^[44]^

The PSN comprises 35 items: 26 assessing logistical aspects (score range 0–78) and 9 assessing interpersonal relationships (score range 9–45). The total PSN score ranges from 9 to 123, with higher scores indicating greater satisfaction with both logistical support and interpersonal relationships. The scale demonstrated excellent internal consistency, with a Cronbach alpha of 0.978.^[48]^

A bilingual expert translated both instruments from English into Arabic. An independent bilingual expert then back-translated the Arabic versions to English. The original and back-translated versions were compared to ensure semantic and conceptual equivalence. To ensure cultural and contextual relevance to the Egyptian healthcare setting, a panel of 5 experts in oncology nursing, nursing education, and instrument development reviewed the Arabic versions. The panel assessed the items for linguistic clarity, cultural appropriateness, and content relevance. Minor wording adjustments were made to improve comprehension among Egyptian nurses and patients without altering the original constructs.

Following expert review, the tools were pilot-tested with a small sample (not included in the main analysis) to assess their clarity and feasibility. Based on the pilot, we made further refinements, particularly to simplify terminology and align with local terminology and healthcare practices. The Content Validity Index (CVI) was calculated during the expert review, and all items met the acceptable threshold for inclusion (CVI ≥ 0.80).

### 2.4. Data collection procedures

Data collection took place from mid-July to the end of August 2024. Initially, the researchers obtained comprehensive lists of nurse navigators, including their work schedules, to support efficient planning. After eligibility screening against predefined criteria, the researchers invited selected nurse navigators to complete a structured questionnaire. Before data collection, the researchers obtained formal ethical approval. The researchers distributed questionnaires in person to nurse navigators during their work hours. The study’s purpose, voluntary nature, and confidentiality assurances were communicated, and the questionnaire’s first page provided detailed information about objectives and ethical considerations. Completing the survey indicated informed consent. Each ONN required approximately 20–25 minutes to complete.

Patient satisfaction data were collected using the PSN, a previously developed instrument that was culturally adapted for use in the present setting. They were offered the option to complete the questionnaire independently, either through face-to-face or telephone interviews. This flexible approach ensured that data collection accommodated patients’ preferences and availability while maintaining reliability and accessibility.

### 2.5. Ethical considerations

The Institutional Review Board of the Faculty of Nursing at Helwan University approved the study (IRB Approval no. 42/13-7-2024). We confirm that our study was conducted in accordance with the principles outlined in the Declaration of Helsinki. All study participants received detailed explanations of the research purpose and the questionnaire content. We obtained written informed consent from each participant before their involvement in the study. Participants were assured that their information would be kept confidential and that all collected data would be used exclusively for scientific research purposes. The voluntary nature of participation was emphasized, and participants were informed that they could refuse to participate or withdraw from the study at any time without consequences. To ensure anonymity, no identifying information was collected, thereby safeguarding participants’ privacy and upholding the ethical integrity of the research process.

### 2.6. Psychometric properties of the study instruments

The study instruments were repurposed for the Egyptian cultural context and assessed for their reliability and validity. After translation into Arabic, 2 instruments were back-translated by a bilingual expert to ensure semantic and conceptual equivalence. Our panel of 5 oncology nursing and nursing education experts reviewed the back-translated versions for linguistic, cultural and content adequacy. The instruments were subsequently refined, if necessary, without affecting the underlying constructs. Both instruments underwent a preliminary test with a sample that was not part of the main study. The CVI was applied to evaluate the extent to which the items in the original instruments represented the necessary and sufficient content for their intended use. All the items satisfied the predefined criterion (CVI ≥ 0.80).

Cronbach alpha was used to measure internal consistency. The ONN Core Competencies Questionnaire achieved excellent overall internal consistency (Cronbach α = 0.95), as well as the domain competencies with 0.91 for “Coordination of Care,” 0.88 for “Communication,” 0.90 for “Education,” 0.85 for “Professional Role,” and 0.92 for “Expert Oncology Nurse Navigator.” The Patient Satisfaction with the Navigator (PSN) Scale demonstrated high internal consistency (Cronbach α = 0.94), with 0.93 for the Logistical Aspects, and 0.89 for the Interpersonal Relationship subscales.

Given that the ONN Core Competencies Questionnaire and the PSN Scale were administered to different respondent populations, their additional psychometric properties were analyzed independently. Joint factor analysis was not performed, and no cross-instrument discriminant validity was assessed.

Due to the small sample size of 65 nurses, compared to the 52-item tool, confirmatory factor analysis (CFA) to evaluate item level construct validity was limited in this study. Therefore, construct validity was assessed at the level of the 5 competency domains. Adequacy of sampling was tested with the Kaiser–Meyer–Olkin (KMO) measure and Bartlett test of sphericity. Factor loadings and explained variance were examined to determine whether the competency constructs were sufficiently represented by the domains. As secondary indicators, composite reliability (CR) and average variance extracted (AVE) were analyzed. These analyses should be interpreted as initial construct validity evidence, rather than complete item level evidence.

For the PSN Scale, in the patient sample, construct validity was analyzed by CFA of the 2 factors: PSN-L and PSN-I. Consideration of overall model fit was based on the Comparative Fit Index (CFI), Tucker–Lewis Index (TLI), root mean square error of approximation (RMSEA) with its 90% confidence interval, and standardized root mean square residual (SRMR). Convergent validity was investigated with standardized factor loadings, CR, and AVE, with CR and AVE set to > 0.70 and > 0.50, respectively. Discriminant validity of PSN-L and PSN-I was evaluated using the heterotrait–monotrait ratio (HTMT) and the Fornell–Larcker criterion.

The ONN Core Competencies Questionnaire demonstrated adequate performance of domain-level constructs. The KMO score was 0.80, and Bartlett test was statistically significant, χ^2^(10) = 155.4, *P* < .001. The 5 competency domains loaded on a common competency factor, and loadings for each domain ranged from 0.73 to 0.84, accounting for 62.5% of the variance. CR and AVE were 0.89 and 0.63, respectively. The relative small sample of nurses led the authors to interpret this evidence as supportive of domain-level constructs, rather than item-level validation.

Regarding the PSN Scale, CFA endorsed the proposed 2-factor structure, χ^2^(559) = 1048.3, *P* < .001, CFI = 0.955, TLI = 0.951, RMSEA = 0.046 (90% CI: 0.042–0.050), and SRMR = 0.051. For PSN-L, factor loadings ranged from 0.62 to 0.80. Ranging from 0.69 to 0.83, factor loadings for PSN-I were somewhat higher. Convergent validity was supported as both CR values were 0.97 and 0.92, respectively, as were the AVE values at 0.52 and 0.58. Discriminant validity was supported with an HTMT value of 0.68 and the Fornell–Larcker criterion as the square root of AVE (0.72 and 0.76) was greater than the correlation among PSN-L and PSN-I (*r* = 0.56).

### 2.7. Data analysis

Data were analyzed using IBM SPSS Statistics version 26 and AMOS. Sociodemographic and professional characteristics of the participants were summarized using descriptive statistics. Categorical variables were presented as frequencies and percentages, whereas continuous variables were summarized using means and standard deviations (M ± SD). Statistical significance was set at *P* < .05.

Independent-samples *t*-tests were used to compare ONN core competency scores and patient satisfaction scores across demographic or professional variables with 2 categories. One-way analysis of variance (ANOVA) was used for variables with 3 or more categories. Significant ANOVA results were followed by Tukey’s honestly significant difference (HSD) post hoc test to identify specific group differences. Effect sizes were reported using Cohen *d* for independent-samples *t*-tests and significant pairwise comparisons.

Before conducting parametric analyses, assumptions of normality, homogeneity of variance, and linearity were assessed. Normality was evaluated using the Shapiro–Wilk test. No significant deviations from normality were observed for ONN competency scores (*W* = 0.972, *P* = .143) or navigator-level mean patient satisfaction scores (W = 0.981, *P* = .392). Homogeneity of variance was assessed using Levene test, with no significant violations identified (*P* > .05). Visual inspection of scatterplots indicated an approximately linear relationship between ONN competency and patient satisfaction scores.

Because ONN competencies and patient satisfaction were reported by different respondent groups, the primary association analysis was conducted at the nurse-navigator level. The study included 65 ONNs and 404 patients. Patients were linked to the navigator responsible for their care, and the satisfaction scores of patients assigned to the same ONN were averaged to generate 1 mean satisfaction score for each navigator. Accordingly, Pearson correlation analysis was based on 65 independent nurse-level paired observations. Pearson correlation coefficients (*r*) and their corresponding 95% confidence intervals were used to evaluate the strength and direction of the associations between ONN competency scores and patient satisfaction.

The number of patients contributing to each navigator-specific mean satisfaction score varied across navigators. Although navigator means based on larger patient clusters may provide more precise estimates of satisfaction, no weighting was applied in the primary cluster-level correlation because the nurse navigator was the unit of analysis and each navigator therefore contributed equally to the analysis. The distribution of patients per navigator was summarized using the mean, SD, median, interquartile range, and range. To assess the potential influence of unequal cluster sizes and the nested structure of patients within nurse navigators, linear mixed-effects modeling and hierarchical bootstrap aggregation were additionally used as sensitivity analyses. Data completeness was assessed before analysis. The final analytic dataset contained no missing item responses among the included questionnaires. Incomplete questionnaires were excluded before analysis; therefore, all 65 ONNs and 404 patients contributed complete data, and no missing-value imputation was required.

Additional psychometric analyses were conducted separately for the 2 culturally adapted instruments because they were administered to different respondent populations. Internal consistency reliability was assessed using Cronbach alpha.

For the ONN Core Competencies Questionnaire, construct validity was explored at the domain level using exploratory factor analysis based on the 5 competency domains. Sampling adequacy was assessed using the KMO measure and Bartlett test of sphericity. Factor loadings and the proportion of explained variance were examined to determine the extent to which the 5 domains represented the broader ONN competency construct. CR and AVE were calculated as supportive indicators of convergent validity, with CR ≥ 0.70 and AVE ≥ 0.50 considered acceptable. Given the relatively small nurse sample (n = 65) in relation to the 52-item instrument, a full item-level CFA was not undertaken, and discriminant validity among the individual ONN competency domains was not formally evaluated. Therefore, the ONN psychometric findings were interpreted as preliminary domain-level evidence rather than complete item-level revalidation.

For the Patient Satisfaction with the Navigator (PSN) Scale, construct validity was evaluated separately within the patient sample using CFA of the hypothesized 2-factor structure comprising the PSN-L and PSN-I subscales. Model fit was assessed using the CFI, TLI, RMSEA with its 90% confidence interval, and SRMR. Convergent validity was evaluated using standardized factor loadings, CR, and AVE, with CR ≥ 0.70 and AVE ≥ 0.50 considered acceptable. Discriminant validity between PSN-L and PSN-I was assessed using the HTMT and the Fornell–Larcker criterion.

## 3. Results

Table 1 provides a comprehensive overview of the study participants, including nurses (n = 65) and patients (n = 404). Among nurses, the majority were under 30 years (70.8%), with a mean age of 26.0 ± 2.03 years. Regarding educational qualifications, 52.3% held a bachelor’s degree, while 47.7% held an associate degree. Most nurses had <5 years of experience (75.4%), with a mean of 3.92 ± 1.08 years. Training sessions were reported as follows: 43.1% attended once, 29.2% twice, and 27.7% 3 times. For patients, the largest age group was those older than 40 years (38.9%), followed by 30 to 40 years (36.4%) and under 30 years (24.8%). Most reported insufficient monthly income (67.3%), while only 32.7% had sufficient income. In terms of residence, 56.4% lived in urban areas, while 43.6% lived in rural areas. Female patients constituted 64.1% of the group, whereas male patients accounted for 35.9%. In addition, each navigator-specific mean satisfaction score was based on 4 to 9 patients, with a mean cluster size of 6.22 ± 1.26 patients (median = 6, IQR = 5–7).

**Table 1:** Characteristics of study subjects.

Characteristics of nurses (n = 65)	n	%
Age		
<30	46	70.8
30–35 Mean ± SD 26.0 ± 2.03	19	29.2
Education level		
Associate degree	31	47.7
Bachelor’s degree	34	52.3
Experience		
<5 yrs	49	75.4
5–10 yrs Mean ± SD 3.92 ± 1.08	16	24.6
Times of training		
Once	28	43.1
Twice	19	29.2
Thrice	18	27.7
Characteristics of patients (n = 404)	n	%
Age		
<30	100	24.8
30–40	147	36.4
>40	157	38.9
Monthly income		
Sufficient	132	32.7
Insufficient	272	67.3
Residence		
Rural	176	43.6
Urban	228	56.4
Gender		
Men	145	35.9
Women	259	64.1
Nurse–patient clustering characteristics		
Total nurse navigators **65**		
Total patients contributing to navigator means **404**		
Patients per navigator, mean ± SD **6.22 ± 1.26**		
Patients per navigator, median (IQR) **6 (5–7**)		
Patients per navigator, range 4–9		

Table 2 presents reliability and descriptive statistics for 2 key variables: ONN core competencies and the PSN Scale. For ONN core competencies, the subscales showed strong internal consistency, with Cronbach α ranging from.85 to.92. Subscale means ranged from 10.80 (SD = 2.19) for “Professional Role” to 19.37 (SD = 2.54) for “Coordination of Care.” The total core competencies score had a Cronbach α of.95 and a mean of 79.28 (SD = 8.24), indicating high reliability and consistent performance across items.

**Table 2:** Reliability and descriptive statistics for oncology nurse navigator core competencies and patient satisfaction scales.

Variables	Mean percent	Cronbach α	Mean ± SD	Min	Max
*Oncology nurse navigator core competencies*					
Coordination of care	74.2%	0.91	19.37 ± 2.54	14	25
Communication	78%	0.88	14.04 ± 1.72	11	17
Education	79.5%	0.90	17.55 ± 2.28	10	22
Professional role	77.1%	0.85	10.80 ± 2.19	5	14
Expert oncology nurse navigator	72.9%	0.92	17.51 ± 3.63	8	24
Total core competencies	76.2%	0.95	79.28 ± 8.24	57	95
*Patient satisfaction with the Navigator Scale*					
Patient satisfaction with logistical aspects	77.3%	0.93	60.28 ± 4.30	50	72
Satisfaction with the interpersonal relationship	73.1%	0.89	32.89 ± 3.62	20	41
Total patient satisfaction with navigation	75.7%	0.94	93.18 ± 5.68	76	112

α = Cronbach alpha, Max = maximum observed score, Min = minimum observed score, SD = standard deviation.

For the PSN Scale, the subscales also demonstrated high reliability, with Cronbach α values of .89 for “Satisfaction with the Interpersonal Relationship” and .93 for “Patient Satisfaction with Logistical Aspects.” The total patient satisfaction score had a Cronbach α of .94 and a mean of 93.18 (SD = 5.68), indicating high patient satisfaction.

Analysis of nurses’ demographic and professional characteristics revealed significant differences in core competency scores (Table 3). Nurses aged 30 to 35 years (M = 82.16, SD = 4.45) had significantly higher competency scores than those younger than 30 years (M = 78.08, SD = 9.15), *t* = −2.41, *P* = .019, with a moderate effect size (*d* = 0.54). Nurses with a bachelor’s degree (M = 81.32, SD = 7.66) also scored significantly higher than those with an associate degree (M = 77.03, SD = 8.39), *t* = −2.16, *P* = .035, *d* = 0.57. The largest difference was observed according to nursing experience; nurses with 5 to 10 years of experience (M = 90.15, SD = 10.30) scored significantly higher than those with <5 years of experience (M = 75.73, SD = 7.62), *t* = −6.01, *P* < .001, with a large effect size (*d* = 1.73). One-way ANOVA also demonstrated significant differences according to training frequency, *F* (2, 62) = 5.82, *P* = .005. Tukey’s HSD post hoc comparisons showed that nurses who had attended training once (M = 75.57, SD = 8.84) scored significantly lower than those who had attended training twice (M = 81.53, SD = 6.86) or 3 times (M = 82.66, SD = 6.43), both *P* < .05, with effect sizes of *d* = 0.74 and *d* = 0.89, respectively. No significant difference was observed between nurses who had attended training twice and those who had attended 3 times.

**Table 3:** Associations between nurses’ demographic characteristics and core competencies.

Characteristics of nurses	Core competencies (Mean ± SD)	*t/F (P*)	Post hoc results (Tukey)	Effect size Cohen *d*
Age				
<30	78.08 ± 9.15	*t* = −2.41 (.019)		*d* = 0.50* (<30 vs 30–35)
30–35	82.16 ± 4.45			
Education level				
Associate degree	77.03 ± 8.39	*t* = −2.16 (.035)		*d* = 0.54* (Associate vs Bachelor)
Bachelor’s degree	81.32 ± 7.66			
Experience				
<5 yrs	75.73 ± 7.62	*t* = −6.01 (<.001)		*d* = 1.73* (<5 vs 5–10 yrs)
5–10 yrs	90.15 ± 10.30			
Times of training				
Once	75.57 ± 8.84	*F* = 5.82 (.005)	Once < Twice (*P* < .05), Once < Thrice (*P* < .05), No sig diff between Twice & Thrice	0.74 (Twice > once) 0.89 (Thrice > once)
Twice	81.53 ± 6.86			
Thrice	82.66 ± 6.43			

*d* = Cohen effect size, *F* = one-way analysis of variance statistic, *p* = probability value, SD = standard deviation, *t* = independent-samples *t*-test statistic.

* Statistical significance was set at *P* < .05.

As shown in Table 4, a one-way ANOVA indicated significant differences in patient satisfaction scores across the 3 age groups, *F *= 31.61, *P* < .001. Tukey’s HSD post hoc comparisons showed that patients younger than 30 years (M = 96.82, SD = 5.54) reported significantly higher satisfaction than those aged 30 to 40 years (M = 93.05, SD = 6.94), *P* < .001, *d* = 0.59, and those older than 40 years (M = 90.72, SD = 5.28), *P* < .001, *d* = 1.13. Patients aged 30 to 40 years also reported significantly higher satisfaction than those older than 40 years, *P* < .001, *d* = 0.38. Patient satisfaction did not differ significantly by monthly income, *t *= 0.59, *P* > .05, or residence, *t *= 0.62, *P* > .05, with negligible effect sizes (*d* = 0.06 for both comparisons). A significant gender difference was observed, with women (M = 94.77, SD = 5.83) reporting higher satisfaction than men (M = 91.13, SD = 4.61), *t *= −6.47, *P* < .001, *d* = −0.67.

**Table 4:** Associations between patient demographic characteristics and patient satisfaction scores.

Characteristics of patients	Patient satisfaction Mean ± SD	*t/F (P*)	Post hoc results (Tukey)	Effect size Cohen *d*
Age				
<30	96.82 ± 5.54	*F* = 31.61 (.002)	*<30 vs 30–40 P < .001*	*0.59*
30–40	93.05 ± 6.94		*<30 vs > 40 P < .001*	*1.13*
>40	90.72 ± 5.28		*30–40 vs > 40 P < .001*	*0.38*
Monthly income				
Sufficient	93.43 ± 6.35	*t* = 0.59 (>.05)		*0.06*
Insufficient	93.07 ± 5.35			
Residence				
Rural	93.43 ± 6.35	*t* = 0.68 (>.05)		*0.06*
Urban	93.07 ± 5.35			
Gender				
Men	91.13 ± 4.61	*t* = 6.47 (<.001)		*−0.67*
Women	94.77 ± 5.83			Medium to large effect size (men lower satisfaction than women)

Table 5 demonstrates statistically significant positive associations between all ONN competency domains and navigator-level mean patient satisfaction (*P* < .001 for all domains). The strongest association was observed for total core competencies (*r* = .70, 95% CI [.55–.81]), followed by the Expert ONN domain (*r* = .65, 95% CI [.50–.78]) and Coordination of Care (*r* = .56, 95% CI [.37–.71]). Sensitivity analyses produced consistent findings, with a standardized association of β = .52 (95% CI [.27–.77], *P* < .001) in the linear mixed-effects model and a hierarchical bootstrap correlation of *r* = .67 (95% CI [.48–.79], *P* < .001), supporting the robustness of the primary nurse-level association.

**Table 5:** Correlation between oncology nurse navigator core competency domains and total patient satisfaction.

Domains	Patient satisfaction (*r*)	95% CI	*P* value
Coordination of care	0.56	0.37–0.71	<.001
Communication	0.44	0.22–0.62	<.001
Education	0.42	0.20–0.60	<.001
Professional role	0.49	0.28–0.66	<.001
Expert oncology nurse navigator	0.65	0.50–0.78	<.001

Sensitivity analyses for total competency score			
Analysis	Estimate	95% CI	*P* value
Cluster-level Pearson correlation	*r* = 0.70	0.55–0.81	<.001
Linear mixed-effects model	Standardized β = 0.52	0.27–0.77	<.001
Hierarchical bootstrap aggregation	*r* = 0.67	0.48–0.79	<.001

CI = confidence interval, *p* = probability value, *r* = Pearson correlation coefficient. Statistical significance was set at *P* < .05. The cluster-level analysis was based on 65 navigator-level observations. The mixed-effects model included 404 patients nested within 65 nurse navigators. Bootstrap estimates were based on 5000 hierarchical resamples. ICC = .26.

## 4. Discussion

The research examined the correlation between nurse navigators’ competencies and patient satisfaction in oncology care. We observed a significant level of competence among ONNs and a high patient satisfaction score. These results corroborate findings from research in various contexts, in which a substantial percentage of nurses reported elevated levels of competence^[49,50]^ and patient satisfaction with ONN core competencies. The high level of nurse navigators’ competencies suggests that the ONN role has become increasingly specialized, with enhanced training and experience being associated with stronger performance and potentially more positive patient experiences.

Among the 5 competency subdomains, education had the highest average score, followed by communication, professional role, and coordination of care, whereas the expert ONN domain had the lowest score. This finding may reflect the advanced expectations associated with expert practice, including clinical expertise, critical thinking, leadership, and autonomy in managing complex cancer care. Limited experience, institutional support, role clarity, and career development opportunities may also contribute to lower self-assessed expert competence. These findings highlight the importance of continuing professional development, mentorship, and clear career-progression pathways for ONNs.

The results indicated a strong positive correlation between overall ONN competencies and navigator-level mean patient satisfaction, with consistent associations at the level of competency subdomains. Similar findings have been reported in other oncology navigation settings.^[33,51]^ A systematic review also found that nurse navigation improved care coordination and patient satisfaction, particularly with a focus on communication.^[27]^ In this regard, one study has been documented in which 1593 adult participants showed that those participants supported by higher-rated navigators reported greater satisfaction with cancer-related care.^[52]^ This finding is consistent across different healthcare settings and may reflect the contribution of communication and care coordination to patient satisfaction and education. In this case, the finding should be interpreted as a specific correlation between ONN competency scores and mean satisfaction ratings of patients assigned to each navigator. Due to the aggregation of patient satisfaction ratings, this finding should not be interpreted to demonstrate that the same association exists for each individual patient. Moreover, because the study has a cross-sectional design, no cause and effect relationship can be established.

Nurses’ age, education, experience, and training frequency were associated with ONN competency scores. This finding is consistent with evidence that nurse navigation programs are valued by patients and healthcare professionals^[5]^ and that ONNs contribute to communication, continuity, timely initiation of treatment, and coordinated care.^[14]^ Age, education, experience, and training may reflect differences in accumulated knowledge, confidence, and professional judgment; however, these cross-sectional associations should not be interpreted as causal effects. Further research should examine how education, experience, and specialized training relate to the development of ONN competencies.

The associations between patients’ age, gender, and satisfaction levels identified in this study are supported by existing evidence showing that demographic characteristics can shape patients’ perceptions and responses to healthcare services. The comprehensive review indicates that nurse navigation has been linked to improved treatment adherence and higher satisfaction; 87% of trials reported higher satisfaction, reinforcing this assertion.^[35]^ The age- and gender-based disparities in satisfaction may reflect variations in health views, communication preferences, and support needs across demographic groups. For instance, older patients may prioritize individualized instruction and reassurance, whereas gender disparities may reflect differing expectations or experiences within the healthcare system. The absence of significant associations between satisfaction and monthly income or residence suggests that satisfaction levels did not differ by these characteristics in the present sample. However, this finding should not be interpreted as evidence that nurse navigation overcomes socioeconomic or geographic barriers. Navigation services should remain responsive to patients’ demographic characteristics, communication preferences, and support needs.

## 5. Conclusion

This study identified a strong positive association between oncology ONN core competencies and patient satisfaction in an Egyptian oncology care setting. Among the competency domains, Expert ONN competency and coordination of care demonstrated the strongest positive associations with navigator-level mean patient satisfaction. Nurse age, education, experience, and training frequency were associated with competency levels, whereas patient age and gender were associated with satisfaction. These findings highlight the relevance of ONN competencies to patient-centered oncology care but should not be interpreted as evidence of causal effects given the cross-sectional design. Continued professional development and institutional support for nurse navigation warrant consideration, while longitudinal and multicenter studies using objective competency assessments are needed to clarify temporal relationships and examine whether changes in navigator competencies are followed by changes in patient satisfaction and clinical outcomes.

### 5.1. Limitations of the study

This study has several limitations. First, its descriptive cross-sectional design does not establish temporal or causal relationships between ONN competencies and patient satisfaction; therefore, the observed association should be interpreted cautiously and should not be considered evidence of causality. Second, ONN competencies were self-assessed and may have been affected by overestimation and social desirability bias. Although competency and satisfaction data were obtained from different respondent groups, reliance on self-reported measures may still introduce method-related response bias. Third, conducting the study at a single Egyptian cancer center limits the generalizability of the findings to other institutions and healthcare settings. Future multicenter, longitudinal studies should incorporate objective or supervisor-rated competency assessments. Although patient satisfaction scores were aggregated at the nurse navigator level, this approach may reduce variability among individual patient experiences. Future studies using larger samples and multilevel modeling approaches are recommended to further examine nurse–patient level relationships. Future studies with larger multicenter samples should consider multivariable regression or multilevel modeling to account for potential confounding variables and further evaluate the independent association between ONN competencies and patient satisfaction.

In addition, the psychometric evaluation of the ONN Core Competencies Questionnaire was limited by the relatively small nurse sample (n = 65) compared with the 52-item instrument, which precluded a robust item-level exploratory or CFA. Therefore, construct and convergent validity findings for the ONN instrument were evaluated at the domain level and should be interpreted as supportive and preliminary rather than as a complete item-level revalidation. Discriminant validity among the individual ONN competency domains was not formally evaluated and should be examined in larger samples. Although the PSN Scale was evaluated separately in the patient sample using item-level CFA, further studies with larger and more diverse samples are recommended to confirm the factorial structure and assess measurement invariance of both culturally adapted instruments across relevant subgroups and settings. Additionally, aggregating individual patient satisfaction ratings to the navigator level introduces aggregation bias, and may allow for ecological fallacy. For this reason, the association at the nurse level in this study must not be interpreted as showing a competency and satisfaction association at the individual patient level. It is suggested that future studies employ multilevel models to study the association of nurses and patients simultaneously.

## Acknowledgments

The authors express their gratitude to Princess Nourah bint Abdulrahman University Researchers Supporting Project number (PNURSP2026R312), Princess Nourah bint Abdulrahman University, Riyadh, Saudi Arabia.

## Author contributions

**Conceptualization:** Hasan Ahmed Awad Bassyouny, Hossam Ali Ismail, Mohamed Ahmed Aly, Mohamed Hashem Kotp.

**Data curation:** Hasan Ahmed Awad Bassyouny, Hossam Ali Ismail, Aliaa Ezz Eldin Abd Elmoaty, Mohamed Ahmed Aly.

**Funding acquisition:** Wafa Almegewly.

**Investigation:** Wafa Almegewly, Aliaa Ezz Eldin Abd Elmoaty, Hossam Ali Ismail.

**Methodology:** Rasha Mohammed Hussien, Samar Mahmoud Mohamed Elhadary, Rasha Kadri Ibrahim, Abdelaziz Hendy.

**Project administration:** Mohamed Ahmed Aly.

**Validation:** Aliaa Ezz Eldin Abd Elmoaty.

**Writing – original draft:** Hasan Ahmed Awad Bassyouny, Hossam Ali Ismail, Aliaa Ezz Eldin Abd Elmoaty, Rasha Kadri Ibrahim, Abdelaziz Hendy, Samar Mahmoud Mohamed Elhadary, Mohamed Hashem Kotp.

**Writing – review & editing:** Mohamed Ahmed Aly, Rasha Kadri Ibrahim, Abdelaziz Hendy, Wafa Almegewly, Rasha Mohammed Hussien, Mohamed Hashem Kotp.
